# Contemporary Strategies for the Synthesis of Tetrahydropyran Derivatives: Application to Total Synthesis of Neopeltolide, a Marine Macrolide Natural Product

**DOI:** 10.3390/md14040065

**Published:** 2016-03-25

**Authors:** Haruhiko Fuwa

**Affiliations:** Graduate School of Life Sciences, Tohoku University, 2-1-1 Katahira, Aoba-ku, Sendai 980-8577, Japan; hfuwa@m.tohoku.ac.jp; Tel.: +81-22-217-6214

**Keywords:** synthetic methods, tetrahydropyrans, oxocarbenium ions, hetero-Diels-Alder cycloadditions, oxa-Michael reactions, palladium-catalyzed alkoxycarbonylations, oxymercurations, radical cyclizations, ring-closing metathesis

## Abstract

Tetrahydropyrans are structural motifs that are abundantly present in a range of biologically important marine natural products. As such, significant efforts have been paid to the development of efficient and versatile methods for the synthesis of tetrahydropyran derivatives. Neopeltolide, a potent antiproliferative marine natural product, has been an attractive target compound for synthetic chemists because of its complex structure comprised of a 14-membered macrolactone embedded with a tetrahydropyran ring, and twenty total and formal syntheses of this natural product have been reported so far. This review summarizes the total and formal syntheses of neopeltolide and its analogues, highlighting the synthetic strategies exploited for constructing the tetrahydropyran ring.

## 1. Introduction

Marine natural products, mainly the secondary metabolites of marine microorganisms, have been a rich source of structurally diverse and biologically intriguing compounds that are potentially useful for dissecting complex cellular biological events at the molecular level [1,2,3,4,5,6]. Moreover, several marine natural products have recently been developed into clinically approved drugs, including trabectedin and eribulin mesylate, and more are currently under clinical trials [7,8,9]. It is more than likely that marine natural products will continuously have significant impact on chemical biology and drug discovery in the forthcoming decades. However, most marine natural products are not abundantly available from natural sources and marine microorganisms are in many cases difficult to cultivate on a large scale. The scarcity of marine natural products oftentimes precludes their in-depth biological investigations and, currently, total synthesis appears to be the only practical means to overcome the material supply problem at the basic research level. An additional bonus of total synthesis is that it enables manipulation of the biological functions of natural products through structural alterations. Structure-function analyses are important for understanding the mode-of-interactions of natural products with biomolecular targets, given that many natural products are actually multifaceted ligands interacting with multiple biomolecules [10]. Since structurally complex marine natural products are intractable to selective functionalizations, total synthesis lays strong foundation for drug discovery research which in many cases requires structural optimization studies. These beneficial aspects of total synthesis justify its significance in marine natural products chemistry and chemical biology.

**Figure 1:**
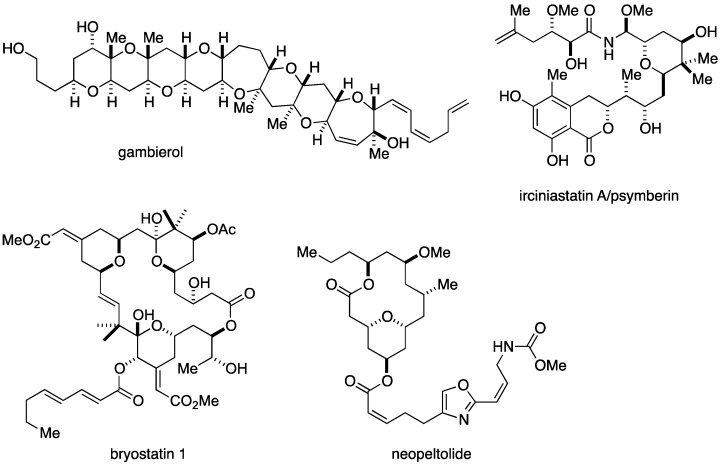
Structures of gambierol (**1**), irciniastatin A (**2**), bryostatin 1 (**3**), and neopeltolide (**4**).

Tetrahydropyrans are structural motifs that are abundantly present in a plethora of biologically active marine natural products. Several representative examples are shown in Figure 1. Gambierol (**1**) was isolated by Satake *et al.* from cultured cells of the ciguatera causative dinoflagellate *Gambierdiscus toxicus* and structurally characterized by extensive NMR analyses and a modified Mosher analysis [11,12]. This compound inhibits voltage-gated potassium ion channels (Kv channels) in a subtype-selective manner at nanomolar concentrations [13,14,15,16] and shows potent acute neurotoxicity in mice. Recently, the Botana group reported that the Kv channel inhibitory activity of gambierol resides in the right-half of the molecule and that gambierol and its truncated analogues reduced the levels of extra- and intracellular amyloid β and hyperphosphorylated tau in an *in vitro* model of Alzheimer’s disease [17,18].

Irciniastatin A (**2**) was isolated by Pettit and co-workers from the marine sponge *Ircinia ramosa* [19]. Almost simultaneously, Crews *et al.* reported the isolation of psymberin from the marine sponge *Psammocinia* sp. [20]. Although the original stereochemical assignments made independently by Pettit and Crews were conflicting, irciniastatin A and psymberin were ultimately shown to be identical through total synthesis by De Brabander and co-workers [21]. Irciniastatin A inhibits protein synthesis and shows a highly potent and selective growth inhibitory effect in human solid tumor cells. A forward genetic screen in *Caenorhabditis elegans* has shown that iriciniastatin A primarily targets the ribosome [22]. A recent report has shown that irciniastatin A induces a potent and sustained activation of the extracellular signal-regulated kinase (ERK) to promote the ectodomain shedding of tumor necrosis factor (TNF) receptor in the A549 human non-small cell lung adenocarcinoma cell line [23].

Bryostatin 1 (**3**), a representative marine macrolide natural product that contains tetrahydropyran rings as substructures, was isolated by the Pettit group from the marine bryozoan *Bugula neritina* and structurally identified by an X-ray crystallographic analysis [24]. More than 20 natural congeners have been identified to date [25]. Bryostatin 1 is a potent and isoform-selective protein kinase C activator and shows anti-cancer, anti-Alzheimer’s disease, and anti-HIV activities. Because of these significant biological activities and synthetically challenging complex molecular architecture, a number of renowned synthetic chemists devoted their efforts to the development of concise synthesis of bryostatin 1 and its congeners [26]. Furthermore, Wender *et al.* reported function-oriented synthesis [27] of structurally simplified bryostatin analogues (bryologs) and their structure-function relationships [28,29,30].

Neopeltolide (**4**), a new member of marine macrolide natural products, was identified by Wright and co-workers from a deep-water sponge of the family Neopeltidae, collected off the north coast of Jamaica [31]. The gross structure including the relative configuration was proposed on the basis of extensive 2D-NMR analyses. Later, total syntheses by Panek *et al.* [32]. and Scheidt *et al.* [33,34] partially reassigned the relative configuration and established the absolute configuration. The structure of neopeltolide resembles with that of leucascandrolide A, another marine macrolide natural product isolated from a calcareous sponge collected along the east coast of New Caledonia [35]. Wright reported that neopeltolide showed potent antiproliferative activity against several human cancer cell lines at nanomolar concentrations. The Kozmin group has reported that neopeltolide specifically inhibits the complex III of the mitochondrial electron transport chain [36]. Fuwa *et al.* have recently described the apoptosis-inducing activity of a synthetic analogue of neopeltolide in HL-60 human promyelocytic leukemia cells [37]. However, the mode-of-action of this natural product have not been fully elucidated. The intriguing structure and potent biological activities of neopeltolide have attracted considerable attention from the synthetic community, and twenty total and formal syntheses of this natural product have been reported so far [38,39,40,41,42,43,44,45,46,47,48,49,50,51,52,53,54,55,56,57,58]. Furthermore, several groups have reported the structure-activity relationships of neopeltolide by evaluating the antiproliferative activity of synthetic analogues against cancer cells [34,43,47,56,59,60]. These efforts have culminated in the elucidation of the pharmacophoric structural elements for potent antiproliferative activity [56].

Total synthesis of these complex marine natural products requires efficient and stereoselective construction of tetrahydropyran rings. As such, the synthetic community put significant efforts to the development of methods for the stereoselective synthesis of tetrahydropyran derivatives [61,62]. In the past years, neopeltolide has served as an ideal target compound for synthetic chemists to showcase their creativity in how to construct the tetrahydropyran ring in an efficient and stereoselective manner. This review summarizes the total/formal syntheses of neopeltolide, highlighting the synthetic strategies exploited for constructing the tetrahydropyran ring [63,64].

## 2. Synthesis of Tetrahydropyrans via Oxocarbenium Ions

As exemplified by the Prins reaction and its variants, reactions that involve an intramolecular addition of an alkene to an oxocarbenium ion intermediate have recently evolved to be powerful and versatile means to construct tetrahydropyran rings in complex settings. Actually, several groups have exploited Prins-type reaction, either intramolecularly or intermolecularly, for the synthesis of the tetrahydropyran ring of neopeltolide.

### 2.1. Total Synthesis by the Panek Group

**Figure 2:**
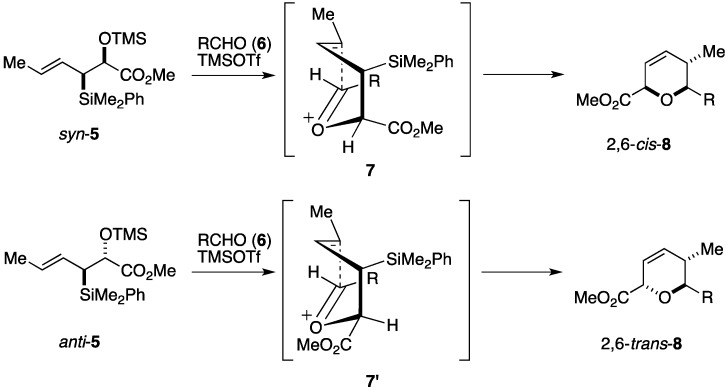
Stereoselective synthesis of dihydropyran derivatives via a formal [4+2]-annulation of chiral crotylsilanes and aldehydes.

Panek and co-workers have developed a versatile method for the synthesis of 2,6-*cis*- and 2,6-*trans*-substituted dihydropyran derivatives by means of a formal diastereoselective [4+2]-annulation of chiral allyl or crotyl silanes and aldehydes [65]. As shown in Figure 2, the reaction begins with coupling of the *syn*-crotylsilane *syn*-**5** with the aldehyde **6** under the influence of a Lewis acid to generate the oxocarbenium ion intermediate **7**. Intramolecular addition of the crotylsilane moiety to the oxocarbenium ion with concomitant loss of the silyl group takes place via a boat-like conformer with maximal σ-p orbital overlap, providing the 2,6-*cis*-substituted dihydropyran derivative 2,6-*cis*-**8**. When the *anti*-crotylsilane *anti*-**5** is used instead, the reaction proceeds via the oxocarbenium ion intermediate **7**′ to deliver the 2,6-*trans*-substituted dihydropyran derivative 2,6-*trans*-**8**. Thus, the stereochemical outcome of the [4+2]-annulation reaction depends on the configuration of the crotylsilane used. The versatility of this synthetic method has been demonstrated in total syntheses of apicularen A [66], bistramide A [67], callipeltoside A [68], kendomycin [69], and leucascandrolide A [70].

The Panek group was the first to show that the original assignment of the relative configuration of neopeltolide was incorrect [32]. Inspection of the available spectroscopic data led them to synthesize a selected set of diastereomers to identify the correct structure. Consequently, Panek *et al.* reassigned the configuration of the C11 and C13 stereogenic centers and at the same time established the absolute configuration as represented by the structure **4**.

**Figure 3:**
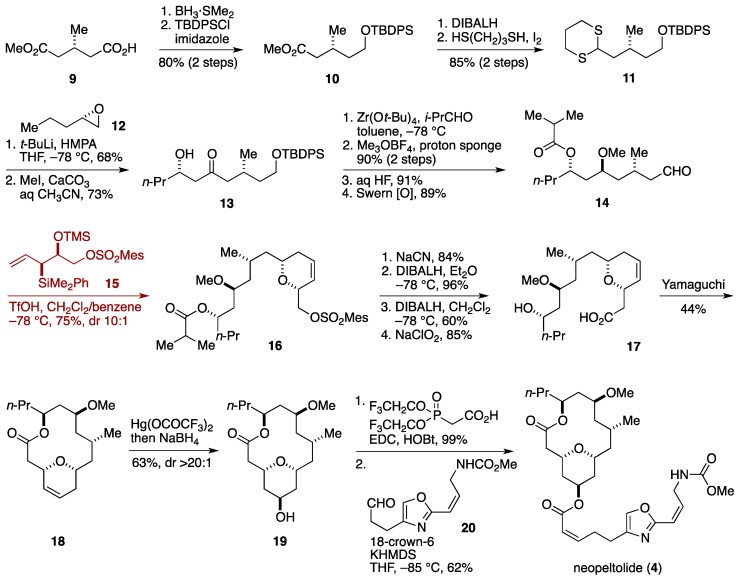
Panek synthesis of neopeltolide.

The Panek synthesis of neopeltolide started with reduction of methyl (*R*)-(+)-3-methylglutarate (**9**) with BH_3_·SMe_2_ to give an alcohol, which was silylated to deliver the TBDPS ether **10** (Figure 3). Half reduction of the ester moiety and dithioacetalization led to the dithiane **11**, which was coupled with the epoxide **12** to afford, after removal of the dithioacetal, β-hydroxy ketone **13**. Modified Evans-Tishchenko reduction [71], *O*-methylation, desilylation, and oxidation [72] provided the aldehyde **14**. The tetrahydropyran ring was forged via a diastereoselective formal [4+2]-annulation of the aldehyde **14** and the allylsilane **15**, providing the dihydropyran derivative **16** in 75% yield with 10:1 diastereoselectivity. The requisite C5 hydroxy group was introduced after the construction of the macrocyclic skeleton. Displacement of the sulfonyl group with NaCN, reductive removal of the acyl group, reduction of the nitrile group, and oxidation [73] of the resultant aldehyde afforded the seco-acid **17**. Yamaguchi macrolactonization [74] of **17** delivered the 14-membered macrolactone **18** in a moderate yield. Oxymercuration of **18** with Hg(OCOCF_3_)_2_ resulted in an axial delivery of the reagent and, after reductive workup with NaBH_4_, the alcohol **19** was isolated in 63% yield with greater than 20:1 diastereoselectivity. Acylation of **19** with bis(2,2,2-trifluoromethyl)phosphonoacetic acid and subsequent Still-Gennari-modified Horner-Wadsworth-Emmons reaction [75] with the aldehyde **20** [76] completed the first total synthesis of neopeltolide (**4**) (19 linear steps from methyl (*R*)-(+)-3-methylglutarate).

### 2.2. Total Synthesis by the Scheidt Group

**Figure 4:**
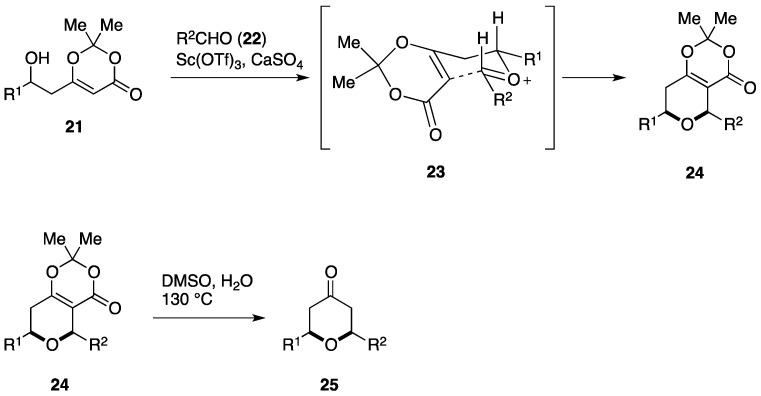
Stereoselective synthesis of 2,6-*cis*-substituted tetrahydropyran-4-one derivatives from β-hydroxy dioxinones.

Scheidt and co-workers have shown that coupling of the β-hydroxy dioxinone **21** with the aldehyde **22** in the presence of Sc(OTf)_3_ gives the oxocarbenium ion intermediate **23**, which subsequently undergoes intramolecular Prins cyclization to afford the bicyclic dioxinone **24** (Figure 4). The stereoselectivity of the Prins cyclization is ascribable to a chair-like transition state. Notably, **24** is readily convertible to the 2,6-*cis*-substituted tetrahydropyran-4-one derivatives **25** simply by heating in DMSO in the presence of H_2_O [77].

**Figure 5:**
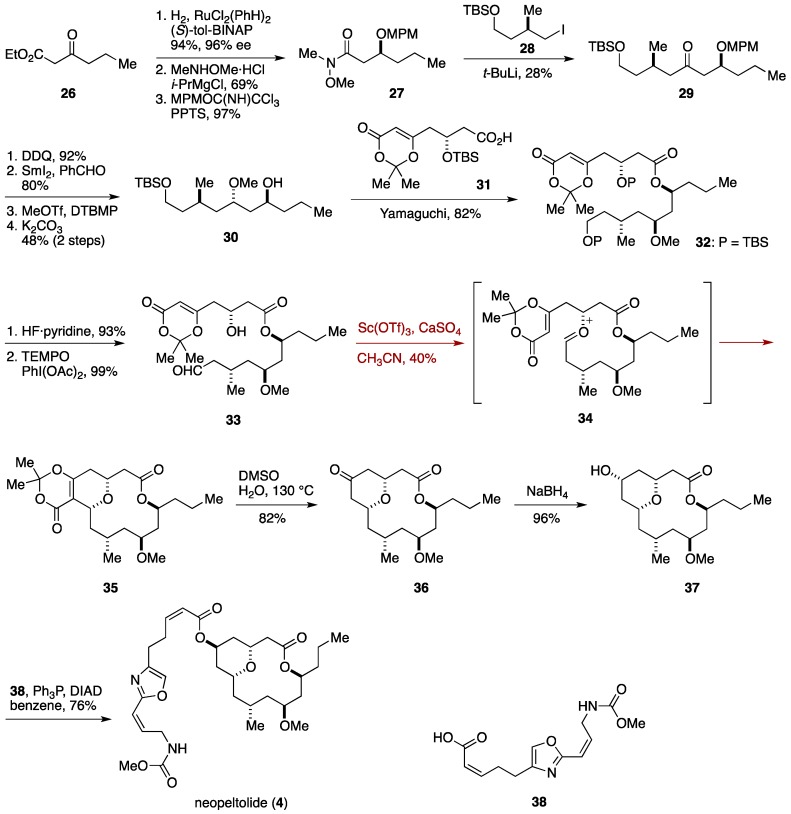
Scheidt synthesis of neopeltolide.

In the Scheidt synthesis of neopeltolide, a Prins-type macrocyclization [78] was successfully implemented to simultaneously construct the tetrahydropyran ring and the macrocyclc skeleton in a single step [33,34]. The synthesis started with Noyori asymmetric hydrogenation [79] of ethyl 3-oxohexanoate (**26**) to deliver the corresponding β-hydroxy ester (Figure 5). Weinreb amidation [80] and protection of the hydroxy group as its MPM ether gave the amide **27**. This was reacted with a lithiation product of the iodide **28** to afford the ketone **29** [81]. Cleavage of the MPM ether, Evans-Tishchenko reduction [82], *O*-methylation, and methanolysis led to the alcohol **30**. The dioxinone **31**, prepared in four steps from 3-(*t*-butyldimethylsilyloxy)propanal via an asymmetric vinylogous Mukaiyama aldol reaction [83], was coupled with the alcohol **30** under Yamaguchi conditions to give the ester **32**. Cleavage of the silyl protecting groups and selective oxidation [84] of the liberated primary alcohol delivered the cyclization precursor **33**. Treatment of **33** with Sc(OTf)_3_ afforded the cyclization product **35** in 40% yield, via the oxocarbenium ion **34**. Thermolysis of **35** in the presence of H_2_O provided the ketone **36**, which was reduced with NaBH_4_ to give neopeltolide macrolactone **37**. Finally, Mitsunobu reaction [85,86] of **37** with the acid **38** [87] furnished neopeltolide (**4**). The significance of this work lies in the demonstration of an intramolecular Prins macrocyclization, which effectively realized a 16-linear step synthetic path to neopeltolide. The Scheidt group further applied their Prins macrocyclization strategy to the total synthesis of exiguolide [88].

### 2.3. Total Synthesis by the Lee Group

**Figure 6:**
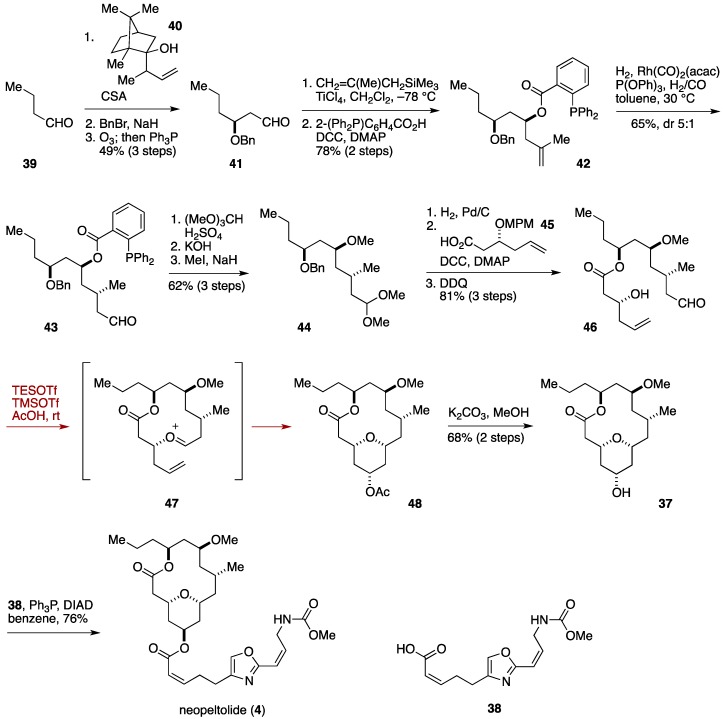
Lee synthesis of neopeltolide.

Shortly after the appearance of the Scheidt synthesis of neopeltolide, another macrocyclic Prins cyclization approach toward neopeltolide was reported by the Lee group, as shown in Figure 6 [38]. The Lee synthesis began with asymmetric crotyl transfer reaction [89] of 1-butanal (**39**) with chiral alcohol **40**. The resultant homoallylic alcohol was benzylated and then subjected to ozonolysis to give the aldehyde **41**. Chelate-controlled methallylation [90] followed by esterification with 2-diphenylphospinobenzoic acid delivered the ester **42**, whose hydroformylation using Rh(CO)_2_(acac)/P(OPh)_3_ catalyst [91] led to the aldehyde **43** in 65% yield with 5:1 diastereoselectivity. After masking the aldehyde moiety, the superfluous 2-diphenylphosphinobenzoyl group was removed to liberate the hydroxy group, which was *O*-methylated to give the methyl ether **44**. Hydrogenolysis of the benzyl ether followed by esterification with the carboxylic acid **45**, and subsequent exposure to DDQ provided the aldehydic homoallylic alcohol **46**. Treatment of **46** with TESOTf/TMSOAc in AcOH at room temperature generated an oxocarbenium ion species **47** that underwent Prins cyclization to afford, after methanolysis of **48**, neopeltolide macrolactone **37** in 68% yield. Mitsunobu reaction of **37** with the carboxylic acid **38** furnished neopeltolide (**4**). The Lee synthesis of neopeltolide was completed in 15 linear steps, harnessing the step-economical aspect of the Prins macrocyclization strategy.

### 2.4. Formal Synthesis by the Maier Group

**Figure 7:**
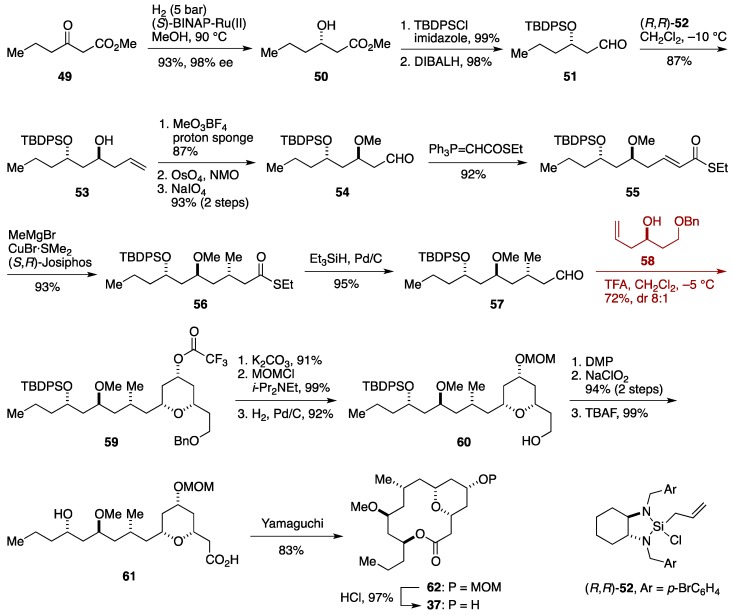
Maier synthesis of neopeltolide.

Maier and co-workers reported the total synthesis of neopeltolide, by exploiting an intermolecular Prins cyclization for the synthesis of the tetrahydropyran ring (Figure 7) [40,43]. The synthesis commenced with Noyori asymmetric hydrogenation of methyl 3-oxobutanoate (**49**) to give the optically active alcohol **50** with excellent enantioselectivity (98% ee), which was silylated and then reduced to provide the aldehyde **51**. Leighton asymmetric allylation [92] of **51** using (*R*,*R*)-**52** delivered the homoallylic alcohol **53**. After *O*-methylation, the terminal olefin was oxidatively cleaved to give the aldehyde **54**, which was treated with Ph_3_P=CHCOSEt to afford the α,β-unsaturated thioester **55**. Diastereoselective conjugate addition of MeMgBr in the presence of CuBr·SMe_2_ and (*S*,*R*)-Josiphos ligand [93] led to the thioester **56**, which was reduced under Fukuyama conditions [94] to provide the aldehyde **57**. This was reacted with the homoallylic alcohol **58** in the presence of TFA to deliver the tetrahydropyran **59** in 72% yield with 8:1 diastereoselectivity. A three-step protecting group manipulations led to the alcohol **60**, which was oxidized [95] and then desilylated to produce the seco-acid **61**. Yamaguchi macrolactonization of **61** afforded the macrolactone **62**. Cleavage of the MOM group led to neopeltolide macrolatone **37**, completing a formal synthesis of neopeltolide (**4**). This formal synthesis proceeded in 19 linear steps from methyl 3-oxohexanoate (**49**).

### 2.5. Total Synthesis by the Kozmin Group

**Figure 8:**
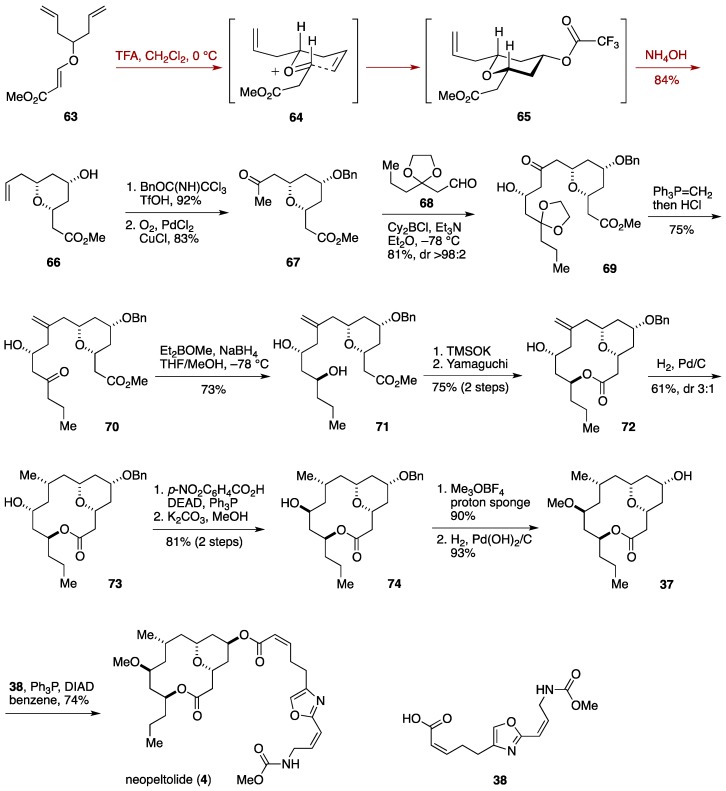
Kozmin synthesis of neopeltolide.

Kozmin and co-workers completed the total synthesis of neopeltolide in a racemic form, highlighting a desymmetrizing Prins cyclization for constructing the tetrahydropyran ring [36]. Treatment of the vinylogous ester **63** with TFA in CH_2_Cl_2_ at 0 °C resulted in Prins cyclization via the oxocarbenium ion **64** to give, after *in situ* cleavage of the trifluoroacetyl group of **65**, the tetrahydropyran derivative **66** in 84% yield (Figure 8). Benzylation of the hydroxy group followed by Wacker oxidation of the olefin provided the methyl ketone **67**. Treatment of **67** with Cy_2_BCl/Et_3_N generated a boron enolate, which was reacted with the aldehyde **68** to deliver the alcohol **69** in 81% yield with excellent stereocontrol (dr > 98:2) [96]. Wittig methylenation of **69** and acidic workup gave the ketone **70**, which was stereoselectively reduced with Et_2_BOMe/NaBH_4_ [97] to afford the 1,3-diol **71**. After hydrolysis of the methyl ester moiety [98], Yamaguchi macrolactonization of the resultant seco-acid led to the macrolactone **72**. Hydrogenation of the exo-methylene of **72** gave the alcohol **73**. Mitsunobu inversion of **73** (*p*-NO_2_C_6_H_4_CO_2_H, Ph_3_P, DEAD) and methanolysis of the resultant *p*-nitrobenzoate delivered the alcohol **74**. *O*-Methylation of **74** was followed by hydrogenolysis of the benzyl ether to afford the neopeltolide macrolactone **37**. Mitsunobu coupling of **37** with the carboxylic acid **38** provided neopeltolide (**4**). A concise synthesis of neopeltolide in a racemic form was thus achieved in 15 linear steps from a commercially available material.

### 2.6. Formal Synthesis by the Floreancig Group

**Figure 9:**
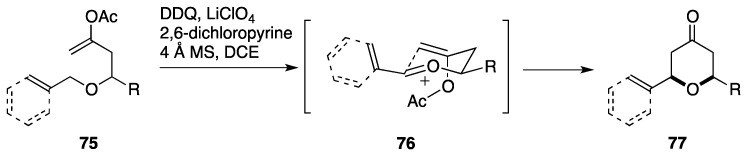
DDQ-mediated intramolecular Prins cyclization.

Floreancig *et al.* have devised a DDQ-mediated oxocarbenium ion generation/intramolecular Prins cyclization for concise synthesis of 2,6-*cis*-substituted tetrahydropyran-4-one derivatives (Figure 9) [99]. The synthetic strategy requires appropriate elaboration of cyclization precursors; upon oxidation of an allylic or benzylic ether moiety of **75** with DDQ, the corresponding oxocarbenium ion **76** is generated, to which an enol acetate moiety attacks intramolecularly with loss of the acetyl group to provide the 2,6-*cis*-substituted tetrahydropyran-4-one derivative **77**. The stereoselectivity can be reasoned by considering a chair-like transition state, as shown. The present synthetic strategy has been applied to the total synthesis of clavosolide A [100].

**Figure 10:**
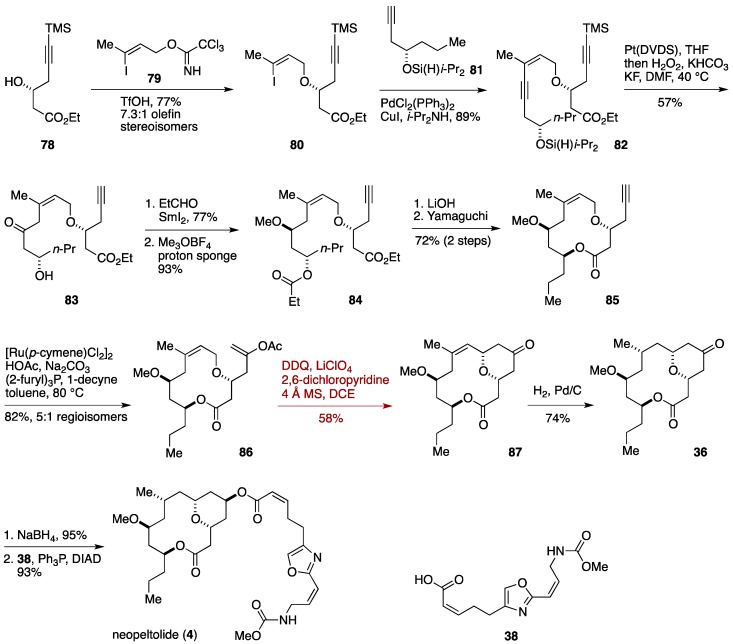
Floreancig total synthesis of neopeltolide.

The Floreancig group has successfully applied their DDQ-mediated intramolecular Prins cyclization strategy to neopeltolide synthesis (Figure 10) [44]. The synthesis began with etherification of optically active alcohol **78**, prepared in one step from a commercially available material, with the trichloroacetimidate derivative **79** under the influence of TfOH to give the vinyl iodide **80**, which was coupled with the alkyne **81** under Sonogashira conditions [101,102] to provide the endiyne **82**. Intramolecular hydrosilylation of **82** catalyzed by Pt(DVDS) followed by oxidation of the derived vinyl silane afforded the hydroxy ketone **83** in 57% yield in a regioselective manner [103]. Evnas-Tishchenko reduction and *O*-methylation led to the methyl ether **84**. Hydrolysis of the ester moieties of **84** gave a seco-acid, which underwent Yamaguchi macrolactonization to deliver the macrolactone **85**. Addition of AcOH to the terminal alkyne was catalyzed by [Ru(*p*-cymene)Cl_2_]_2_ to afford the enol acetate **86** (82% yield, 5:1 regioselectivity) [104]. Exposure of **86** to DDQ in the presence of 2,6-dichloropyridine and LiClO_4_ in 1,2-dichloroethane induced the expected oxocarbenium ion generation/intramolecular Prins cyclization sequence to provide the ketone **87** in 58% yield. Stereoselective hydrogenation of **87** furnished known compound **36**, which intercepts the Scheidt synthesis of neopeltolide. The Floreancig formal synthesis was completed in 13 linear steps from 2-butyn-1-ol.

### 2.7. Formal Synthesis by the Yadav Group

**Figure 11:**
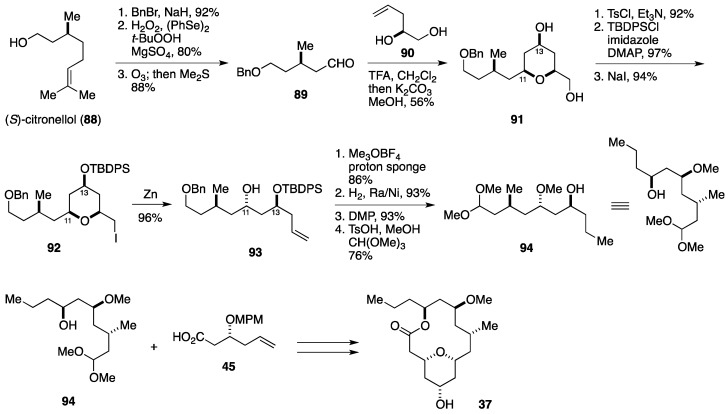
Yadav formal synthesis of neopeltolide.

Yadav and Kumar described a formal synthesis of neoleptolide based on Lee’s intramolecular Prins macrocyclization strategy (Figure 11) [48]. The Yadav synthesis started with conversion of (*S*)-citronellol (**88**) into the aldehyde **89**, whose intermolecular Prins cyclization with the homoallylic alcohol **90** gave the tetrahydropyran derivative **91** (56% yield), where the C11 and C13 stereogenic centers were established. A three-step functional group manipulation led to the iodide **92**. The terahydropyran ring of **92** was cleaved by treatment with zinc to deliver the alcohol **93**, which was elaborated to the alcohol **94** in four steps. The remainder of the synthesis was achieved in the same way as that reported by Lee *et al.* [38].

### 2.8. Formal Synthesis by the Jennings Group

**Figure 12:**
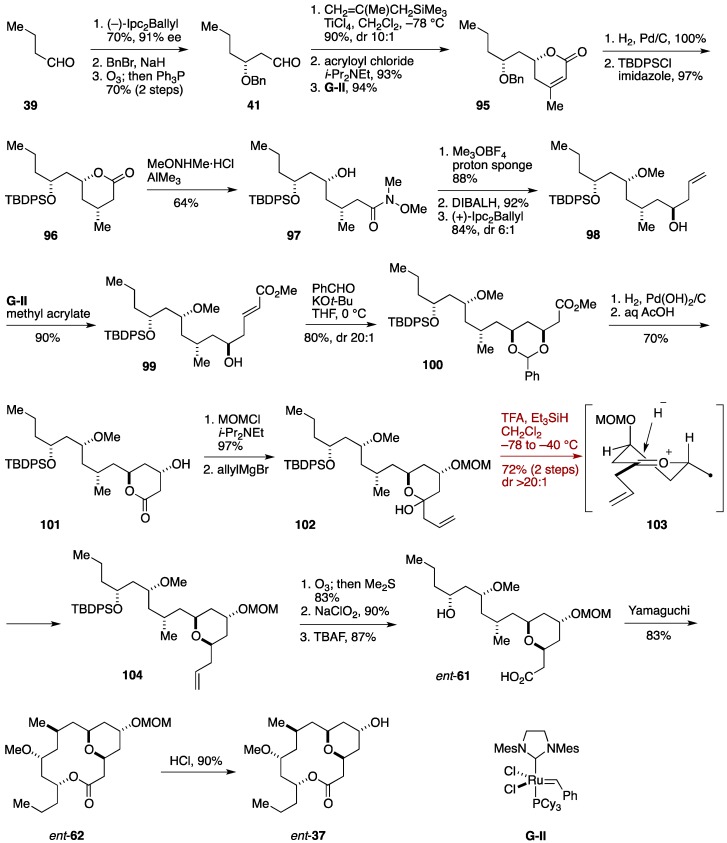
Jennings formal synthesis of neopeltolide.

Martinez-Solorio and Jennings reported a formal synthesis of (−)-neopeltolide, the enantiomer of the natural product, by using an oxocarbenium ion formation/stereoselective reduction of a six-membered hemiacetal derivative (Figure 12) [51]. Stereoselective tetrahydropyran synthesis by means of reduction of six-membered hemiacetals with Et_3_SiH in the presence of a Lewis acid was originally developed by Kishi *et al.* [105]. The synthesis commenced with Brown asymmetric allylation [106] of 1-butanal (**39**) to give a homoallylic alcohol, which was benzylated and then ozonolyzed to deliver the aldehyde **41**. Chelate-controlled methallylation of **41**, acylation with acryloyl chloride, and ring-closing metathesis using the second-generation Grubbs catalyst (**G**-**II**) [107] provided the α,β-unsaturated lactone **95**. Stereoselective hydrogenation/hydrogenolysis and silylation gave the lactone **96**, which was reacted with an aluminum amide prepared from MeONHMe·HCl/AlMe_3_ to produce the amide **97**. *O*-Methylation, DIBALH reduction, and Brown asymmetric allylation led to the homoallylic alcohol **98**. Olefin cross-metathesis [108] of **98** with methyl acrylate under the influence of **G**-**II** delivered the α,β-unsaturated ester **99**, which was treated with PhCHO and KO*t*-Bu to afford the benzylidene acetal **100** via intramolecular oxa-Michael reaction [109]. Hydrogenolysis of **100** followed by acid treatment provided the β-hydroxy lactone **101**, which was protected and then allylated to give the hemiacetal **102**. Upon treatment of **102** with Et_3_SiH and TFA, the oxocarbenium ion **103** was generated and stereoselectively reduced to furnish the tetrahydropyran derivative **104** in 72% yield with 20:1 diastereoselectivity. Ozonolysis, Pinnick oxidation, and desilylation led to the seco-acid *ent*-**61**. Yamaguchi macrolactonization of *ent*-**61** provided the macrolactone *ent*-**62**, from which the MOM group was removed to afford neopeltolide macrolactone *ent*-**37**. The present formal synthesis proceeded in 24 linear steps from 1-butanal (**39**).

## 3. Synthesis of Tetrahydropyrans via Hetero-Diels-Alder Cycloaddition

Hetero-Diels-Alder cycloaddition of Danishefsky’s dienes and aldehydes under Lewis acid catalysis is an efficient means to access a variety of 2,6-disubstituted tetrahydropyran-4-one derivatives [110]. The synthetic utility of hetero-Diels-Alder cycloaddtion has been greatly improved by the development of Jacobsen’s chiral chromium(III) catalysts [111]. This section illustrates three independent total/formal syntheses of neopeltolide, each featuring a hetero-Diels-Alder cycloaddition for constructing the tetrahydropyran substructure.

### 3.1. Total Synthesis by the Paterson Group

**Figure 13:**
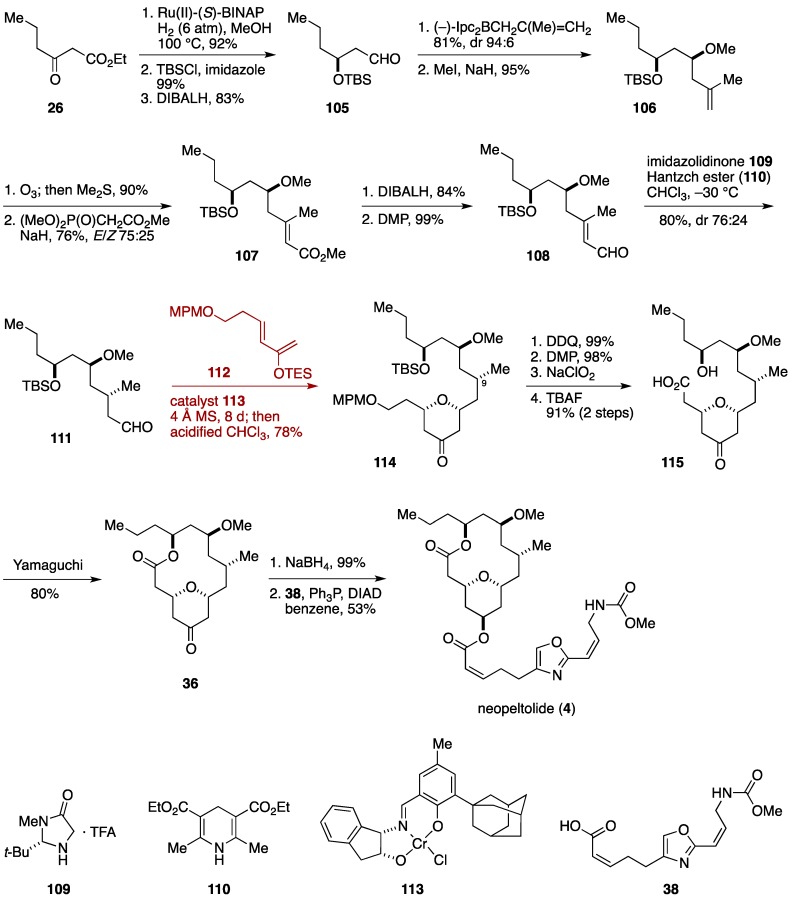
Paterson total synthesis of neopeltolide.

Paterson and Miller implemented a hetero-Diels-Alder cycloaddition in a late stage of their neopeltolide synthesis (Figure 13) [41]. The Paterson synthesis began with Noyori asymmetric hydrogenation of ethyl 3-oxohexanoate (**26**) to give an optically active β-hydroxy ester, which was silylated and then reduced to deliver the aldehyde **105**. Asymmetric methallylation of **105** was followed by *O*-methylation to afford the methyl ether **106**, which was ozonolyzed and then homologated to provide the α,β-unsaturated ester **107**. After oxidation state adjustment, the resultant aldehyde **108** was reduced using the imidazolidinone catalyst **109** and Hantzch ester **110** [112] to give the aldehyde **111** in 80% yield with moderate diastereoselectivity (dr 76:24). Exposure of a mixture of **111** and the 2-silyloxydiene **112** to the Jacobsen’s chiral tridentate chromium(III) catalyst **113** furnished, after mild acidic workup, the 2,6-*cis*-substituted tetrahydropyran-4-one **114** in 78% yield. At this stage, the major diastereomer **114** could be separated from its C9 epimer. Cleavage of the MPM ether, a two-stage oxidation, and desilylation gave the seco-acid **115**. Yamaguchi macrolactonization of **115** provided the macrolactone **36**, which was reduced with NaBH_4_ and then coupled with the carboxylic acid **38** to furnish neopeltolide (**4**). The present synthesis was completed in 18 linear steps from ethyl 3-oxohexanoate (**26**).

### 3.2. Formal Synthesis by the Raghavan Group

**Figure 14:**
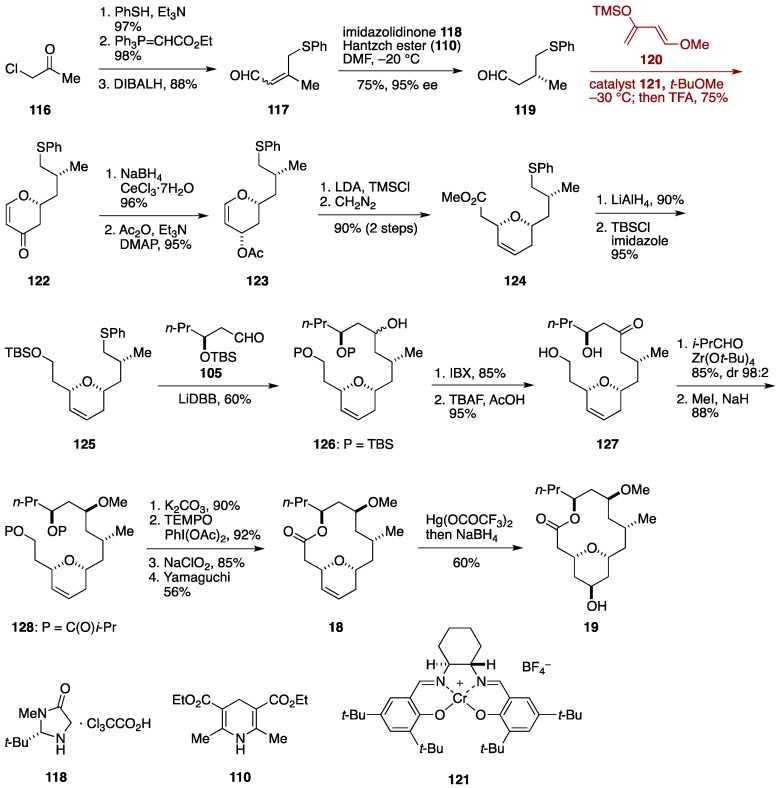
Raghavan formal synthesis of neopeltolide.

In contrast to the Paterson group, Raghavan and Samanta exploited a hetero-Diels-Alder cycloaddition at an early stage of their neopeltolide synthesis (Figure 14) [54]. The Raghavan synthesis started with organocatalytic 1,4-reduction of the α,β-unsaturated aldehyde **117**, prepared in three steps from 2-chloroacetone (**116**), with the imidazolidinone catalyst **118** and Hantzch ester **110** to deliver the aldehyde **119** in 75% yield with excellent enantioselectivity (95% ee). Then, hetero-Diels-Alder cycloaddition with Danishefsky’s diene **120** [113] was undertaken in the presence of (*S*,*S*)-Cr(III)-salen complex **121** [114] to afford, after acidic workup, the dihydropyranone **122** in 75% yield as the only detectable isomer. Luche reduction [115] of **122** followed by acetylation gave the acetate **123**, whose Ireland-Claisen rearrangement [116,117] provided the ester **124**, after esterification with diazomethane. Reduction of **124** with LiAlH_4_ and subsequent silylation delivered the sulfide **125**. Lithiation of **125** with LiDBB [118] followed by addition of the aldehyde **105** produced the coupling product **126** in 60% yield as a mixture of diastereomers. This mixture was oxidized and then desilylated to give the β-hydroxy ketone **127**, which was reduced using the modified Evans-Tishchenko protocol to afford, after *O*-methylation, the diester **128**. Solvolysis of the esters, selective oxidation, and Yamaguchi macrolactonization led to the macrolactone **18**. Finally, oxymercuration of **18** according to Panek and co-workers [32] delivered neopeltolide macrolactone **19**. The present formal synthesis was achieved in 21 linear steps from 2-chloroacetone.

### 3.3. Total Synthesis by the Arun K. Ghosh Group

**Figure 15:**
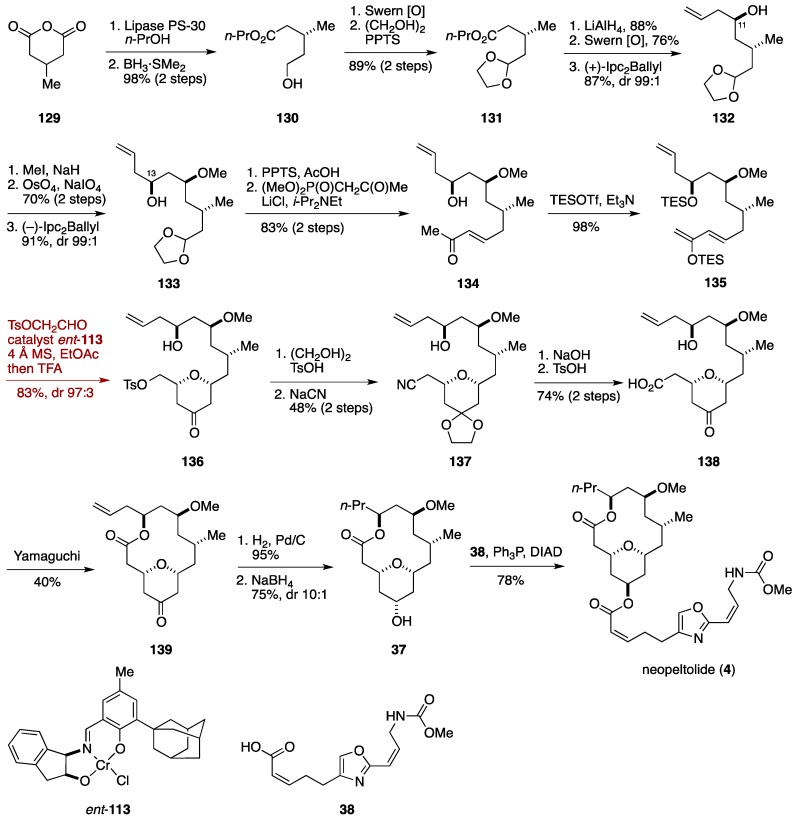
Arun K. Ghosh total synthesis of neopeltolide.

Arun K. Ghosh and co-workers from Purdue University utilized a hetero-Diels-Alder cycloaddition for forging the tetrahydropyran ring at a late stage of their neopeltolide synthesis (Figure 15) [57]. The Arun K. Ghosh synthesis commenced with desymmetrization of 3-methylglutaric anhydride (**129**) with lipase PS-30 to give a carboxylic acid, which was selectively reduced with BH_3_·SMe_2_ to deliver the alcohol **130**. Oxidation of **130** followed by acetalization led to the acetal **131**. Next, the C11 and C13 stereogenic centers were introduced by a two-fold use of Brown asymmetric allylation. Thus, LiAlH_4_ reduction, oxidation, and asymmetric allylation gave the homoallylic alcohol **132**. *O*-Methylation, Lemieux-Johnson oxidation, and the second asymmetric allylation provided the homoallylic alcohol **133**. Deprotection of the acetal of **133** was followed by Horner-Wadsworth-Emmons reaction [119] to deliver the α,β-unsaturated ketone **134**, which was treated with TESOTf/Et_3_N to afford the enol silyl ether **135** with concomitant protection of the free hydroxy group. Hetero-Diels-Alder cycloaddition of **135** with 2-tosyloxyacetaldehyde by the action of Jacobsen’s chiral chromium(III) catalyst *ent*-**113** provided the 2,6-*cis*-substituted tetrahydropyran-4-one **136** in 83% yield with excellent diastereoselectivity (dr 97:3), after acidic workup. Here, the choice of the aldehyde component was critical for the success of the hetero-Diels-Alder cycloaddition. When a one carbon-homologated aldehyde, such as 3-benzyloxypropanal or 3-*t*-butyldimethylsilyloxypropanal, were reacted with the diene **135**, the corresponding cycloaddition product was isolated in only moderate yield. The keto group of **136** was temporarily masked and then the tosyl group was displaced with NaCN to give the cyanide **137**. Hydrolysis of the nitrile group followed by deprotection of the acetal led to the seco-acid **138**, which underwent Yamaguchi macrolactonization to afford the macrolactone **139**. Hydrogenation and NaBH_4_ reduction delivered neopeltolide macrolatone **37**, which was esterified with the carboxylic acid **38** under Mitsunobu conditions to furnish neopeltolide (**4**). The Arun K. Ghosh synthesis was finished in 22 linear steps from 3-methylglutaric anhydride (**129**).

## 4. Synthesis of Tetrahydropyrans via Ring-Closing Metathesis

Olefin metathesis has evolved as an indispensable means for connecting C–C bonds in complex settings because of its remarkable functional group tolerance as well as the commercial availability of various catalysts with a range of reactivity [120,121]. Now, olefin metathesis is an established method for constructing small- to large-membered cyclic alkenes [122,123].

### Total Synthesis by the Fuwa Group (First-Generation Synthesis)

**Figure 16:**
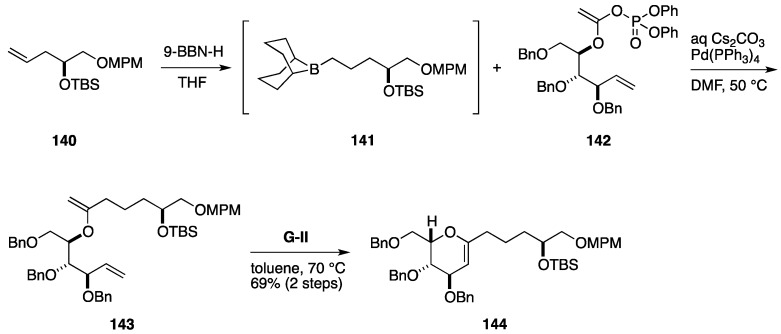
Suzuki-Miyaura coupling/ring-closing metathesis sequence.

Fuwa and co-workers have developed an efficient method for dihydropyran synthesis, in which a Suzuki-Miyaura coupling [124,125,126] and a ring-closing metathesis were exploited as key C–C bond forming reactions [127]. An example is illustrated in Figure 16. Suzuki-Miyaura coupling of the alkylborane **141**, prepared from the olefin **140**, and the enol phosphate **142** under the influence of a base and a Pd catalyst provided the enol ether **143**, which participated in ring-closing metathesis by the action of **G-II** catalyst to afford the dihydropyran **144**. Notably, the product **144** can be readily transformed to synthetically useful derivatives, including tetrahydropyrans and spiroacetals. The present synthetic methodology has been applied to total syntheses of attenol A [127] and gambieric acid A [128,129] and a partial synthesis of okadaic acid [130].

**Figure 17:**
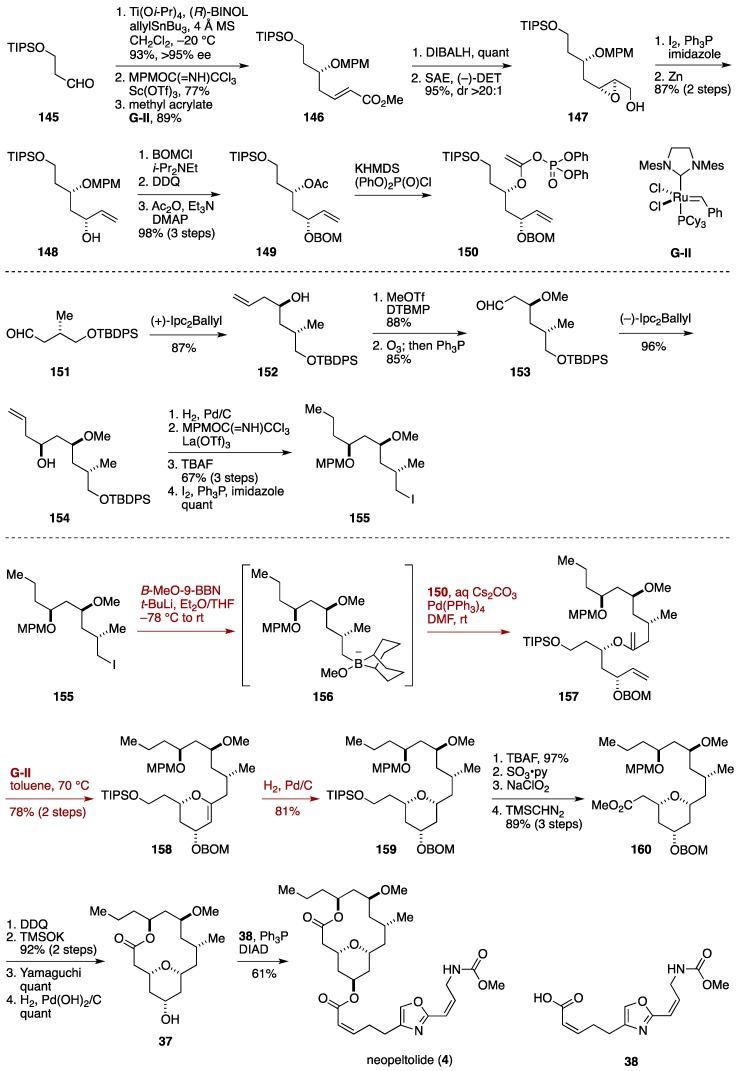
Fuwa total synthesis of neopeltolide.

The Fuwa synthesis started with Keck asymmetric allylation [131] of the aldehyde **145** to give a homoallylic alcohol (93%, >95% ee), which was protected as its MPM ether and then homologated via olefin cross-metathesis to deliver the α,β-unsaturated ester **146** (Figure 17) [39]. DIBALH reduction of **146** provided an allylic alcohol, whose Sharpless asymmetric epoxidation using (−)-DET as a chiral ligand afforded the epoxy alcohol **147** as a single stereoisomer. Iodination and zinc reduction gave the allylic alcohol **148**, setting the C5 stereogenic center. A three-step protecting group manipulation led to the acetate **149**, which was enolized with KHMDS in the presence of (PhO)_2_P(O)Cl to provide the enol phosphate **150**.

Meanwhile, the iodide **155**, the coupling partner of the enol phosphate **150**, was synthesized from known aldehyde **151**. The C11 and C13 stereogenic centers were established through a two-fold application of Brown asymmetric allylation. Thus, asymmetric allylation of **151** with (+)-Ipc_2_Ballyl gave the homoallylic alcohol **152**, which was *O*-methylated and then ozonolyzed to deliver the aldehyde **153**. The second asymmetric allylation using (−)-Ipc_2_Ballyl afforded the homoallylic alcohol **154**. After hydrogenation, the free hydroxy group was masked as its MPM ether [132]. Subsequent desilylation and iodination furnished the iodide **155**.

The Suzuki-Miyaura coupling of the alkylborane **156**, generated from the iodide **155** by lithiation and trapping with *B*-MeO-9-BBN [133], with the enol phosphate **150** was achieved by the action of Pd(PPh_3_)_4_ and Cs_2_CO_3_. The resultant enol ether **157** was subjected to ring-closing metathesis using **G**-**II** catalyst, giving rise to the dihydropyran **158** in 78% yield (two steps). Stereoselective hydrogenation of **158** led to the tetrahydropyran derivative **159** in 81% yield as a single stereoisomer. Desilylation, oxidation [134], and esterification gave the ester **160**. Removal of the MPM group and ester hydrolysis provided a seco-acid, which cleanly underwent Yamaguchi macrolactonization to afford neopeltolide macrolatone **37** after cleaving the BOM ether. Finally, **37** was coupled with the carboxylic acid **38** under Mitsunobu conditions furnished neopeltolide (**4**). The present synthesis proceeded in 24 linear steps from 1,3-propanediol.

## 5. Synthesis of Tetrahydropyrans via Intramolecular Radical Cyclizations

Because of their excellent functional group compatibility and powerful bond-forming ability, radical cyclizations have been exploited in the synthesis of small- and medium-sized ring systems, especially five- and six-membered carbo- and heterocycles [135]. Lee and co-workers have reported that intramolecular radical cyclization of β-alkoxyacrylates is a highly useful method for the stereoselective synthesis of 2,5-*cis*-substituted tetrahydrofurans and 2,6-*cis*-substituted tetrahydropyrans [136].

### Formal Synthesis by the Taylor Group

**Figure 18:**
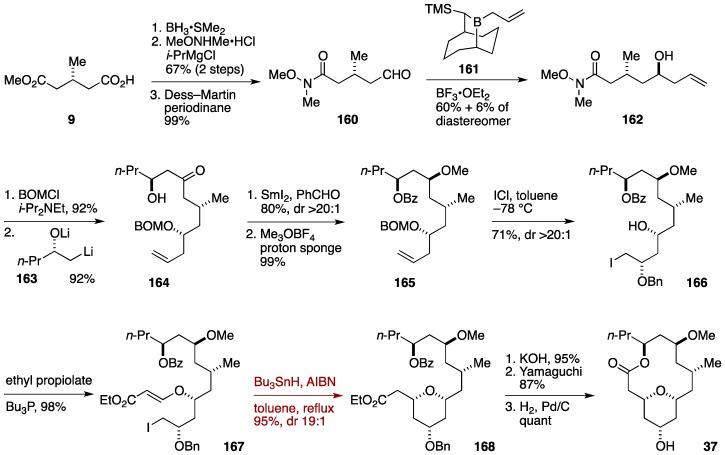
Taylor formal synthesis of neopeltolide.

Taylor and co-workers took advantage of an ether transfer reaction developed in their laboratory [137] and Lee’s radical cyclization methodology for constructing the tetrahydropyran ring of neopeltolide (Figure 18) [42]. The Taylor synthesis began with reduction of methyl (*R*)-(+)-3-methylglutarate (**9**) with BH_3_·SMe_2_, which was followed by Weinreb amidation and Dess-Martin oxidation to give the aldehyde **160**. Asymmetric allylation of **160** using Soderquist’s reagent **161** [138] delivered the homoallylic alcohol **162**. After protection as its BOM ether, the resultant product was reacted with the alkyllithium **163** prepared from the corresponding α-hydroxy sulfide to afford the ketone **164**. Evans-Tishchenko reduction followed by *O*-methylation afforded the methyl ether **165**. Treatment of **165** with ICl resulted in ether transfer reaction to provide the iodide **166** in 71% yield with greater than 20:1 diastereoselectivity. 1,4-Addition of **166** to ethyl propiolate led to the acrylate **167**, whose radical cyclization using *n*-Bu_3_SnH/AIBN in refluxing toluene furnished the tetrahydropyran derivative **168** in 95% yield with 19:1 diastereoselectivity. Thus, the tetrahydropyran ring of neopeltolide was constructed in a highly stereocontrolled manner, by combining an ether transfer reaction and a radical cyclization. Ester hydrolysis, Yamaguchi macrolactonization, and debenzylation afforded neopeltolide macrolactone **37**. The Taylor synthesis was completed in a 14-linear step sequence from methyl (*R*)-(+)-3-methylglutarate (**9**).

## 6. Synthesis of Tetrahydropyrans via Intramolecular Oxa-Michael Reaction

Despite of their moderate nucleophilicity, intramolecular 1,4-addition of alcohols to α,β-unsaturated carbonyl systems (oxa-Michael addition) is known as a useful method for the synthesis of tetrahydropyran derivatives [139,140]. The stereochemical outcome of intramolecular oxa-Michael reaction depends on the local structure and reactivity of substrates and the reaction conditions employed. In general, intramolecular oxa-Michael reaction of ζ-hydroxy α,β-unsaturated esters catalyzed by a base produces 2,6-*trans*-substituted tetrahydropyrans under kinetic conditions, whereas it provides 2,6-*cis*-substituted tetrahydropyrans under thermodynamic conditions [141]. Intramolecular oxa-Michael reaction can also be catalyzed by an acid, provided the conjugate acceptor sufficiently electrophilic [141]. Notably, 2-tetrahydropyranylacetic acid substructures found in many tetrahydropyran-containing natural products might be biosynthetically derived from the corresponding ζ-hydroxy α,β-unsaturated thioesters via intramolecular oxa-Michael reaction [142,143,144].

### 6.1. Formal Synthesis by the Hong Group

**Figure 19:**
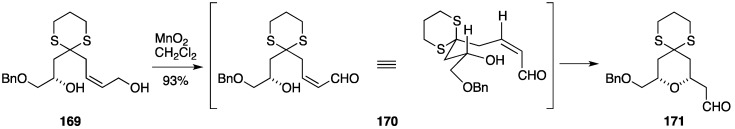
Allylic oxidation/intramolecular oxa-Michael addition cascade.

Hong and co-workers developed an allylic oxidation/intramolecular oxa-Michael addition cascade for the stereoselective synthesis of 2,6-*cis*-substituted tetrahydropyran derivatives [45]. Hong *et al.* focused on the Thorpe-Ingold effect of a dithioacetal group for promoting intramolecular oxa-Michael reaction. An example is shown in Figure 19. Thus, allylic oxidation of the alcohol **169** provided the α,β-unsaturated aldehyde **170**, which readily underwent intramolecular oxa-Michael reaction to deliver the 2,6-*cis*-substituted tetrahydropyran **171**. The stereoselectivity of this cascade reaction can be explained by the conformation of **170**, where the conjugate acceptor is pseudo-equatorially exposed to avoid unfavorable non-bonding interaction with the dithioacetal.

**Figure 20:**
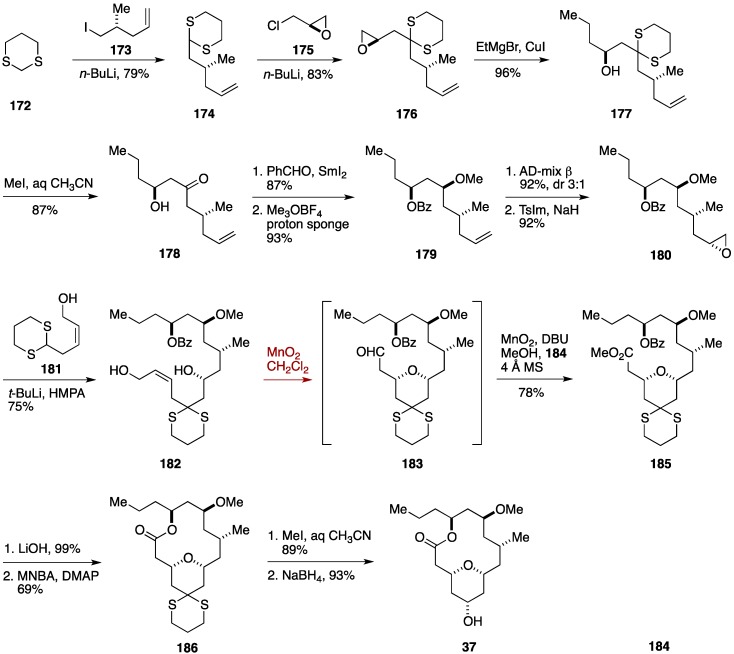
Hong formal synthesis of neopeltolide.

The Hong synthesis commenced with alkylation of 1,3-dithiane (**172**) with (*R*)-5-iodo-4-methylpentene (**173**) [145], prepared in three steps from a commercially available material, to give the dithiane derivative **174**, which was further alkylated with (*R*)-epichlorohydrin (**175**) to deliver the epoxide **176** (Figure 20) [45]. Addition of EtMgBr in the presence of CuI provided the alcohol **177**, from which the dithioacetal was removed to give the β-hydroxy ketone **178**. Evans-Tishchenko reduction followed by *O*-methylation led to the methyl ether **179**, which was dihydroxylated with AD-mix β [146] and then treated with *p*-tosylimidazole/NaH [147] to afford the epoxide **180**. The epoxide **180** was coupled with a lithiated derivative of the dithiane **181** to deliver the triol **182**. Exposure of **182** to MnO_2_ promoted allylic oxidation/intramolecular oxa-Michael addition cascade to furnish the aldehyde **183**, which was *in situ* transformed to the corresponding ester **185** by the action of dimethyltriazolium iodide (**184**), DBU, and MeOH [148]. Hydrolysis of **185** was followed by macrolactonization under Shiina conditions [149] provided the macrolactone **186**. Removal of the dithioacetal and reduction of the resultant ketone afforded neopeltolide macrolactone **37**. The longest linear sequence of the Hong formal synthesis was 17 steps from a commercially available material.

### 6.2. Total Synthesis by the Roulland Group

**Figure 21:**
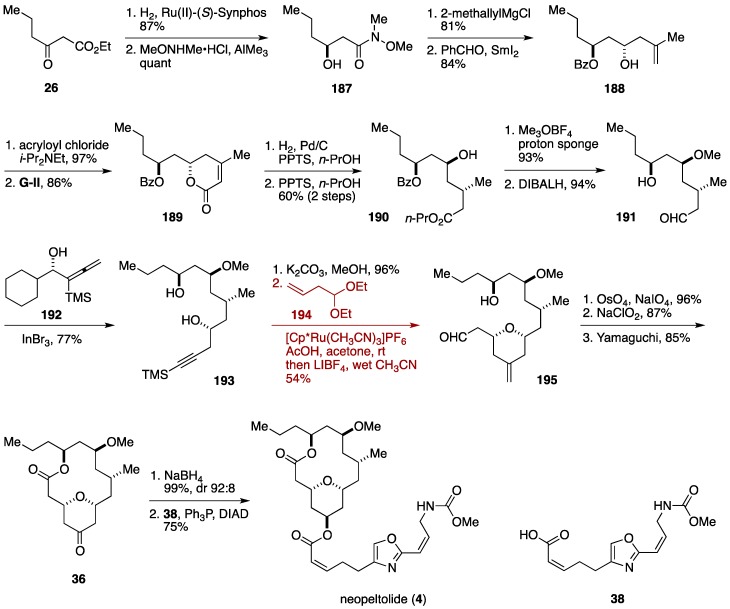
Roulland total synthesis of neopeltolide.

The Roulland group implemented a ruthenium-catalyzed ene-yne coupling/intramolecular oxa-Michael addition cascade in constructing the tetrahydropyran ring of neopeltolide (Figure 21) [46]. This type of cascade reaction was originally developed by Trost and co-workers [150]. The Roulland synthesis began with asymmetric hydrogenation [151] of ethyl 3-oxohexanoate (**26**) to give a β-hydroxy ester, which was converted to the amide **187**. Addition of 2-methallylmagnesium chloride to **187** was followed by Evans-Tishchenko reduction to provide the alcohol **188**. After acylation with acryloyl chloride, ring-closing metathesis of the resultant ester delivered the α,β-unsaturated lactone **189**. Stereoselective hydrogenation and acid-catalyzed solvolysis gave the ester **190**, which was *O*-methylated and then reduced with DIBALH to afford the aldehyde **191**. InBr_3_-catalyzed asymmetric propargylation of **191** using the chiral allenyl alcohol **192** furnished the alcohol **193** [152]. After desilylation, the resultant terminal alkyne was reacted with 4,4-diethoxybut-1-ene (**194**) in the presence of [Cp*Ru(CH_3_CN)_3_]PF_6_ and acetic acid to promote alkyne-enal coupling/intramolecular oxa-Michael addition cascade, giving rise to the 2,6-*cis*-substituted tetrahydropyran derivative **195**, after *in situ* hydrolysis of the diethyl acetal moiety. Addition of a catalytic amount of acetic acid was instrumental in achieving the cascade reaction in an efficient manner. Oxidative cleavage of the exo-methylene, Pinnick oxidation, and Yamaguchi macrolactonization afforded the macrolactone **36**. Finally, NaBH_4_ reduction followed by Mitsunobu reaction with the carboxylic acid **38** furnished neopeltolide (**4**). The Roulland synthesis proceeded in 17 linear steps from ethyl 3-oxohexanoate (**26**).

### 6.3. Total Synthesis by the Fuwa Group (Second-Generation Synthesis)

**Figure 22:**
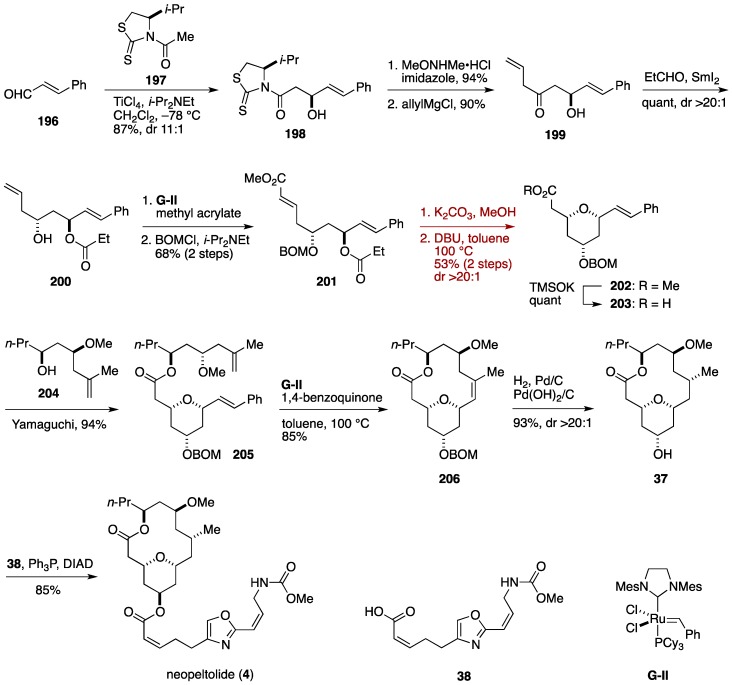
Fuwa total synthesis of neopeltolide.

The Fuwa group disclosed a second-generation synthesis of neopeltolide, featuring an intramolecular oxa-Michael addition of a ζ-hydroxy α,β-unsaturated ester under thermodynamic conditions to forge the tetrahydropyran ring, and a macrocyclic ring-closing metathesis for constructing the 14-membered skeleton (Figure 22) [49,56]. The synthesis started with Nagao asymmetric acetate aldol reaction [153] of *trans*-cinnamaldehyde (**196**) with a titanium enolate of the chiral thiazolidinethione **197** to deliver the alcohol **198** in 87% yield (dr 11:1). After removal of the chiral auxiliary via amidation, addition of allylmagnesium chloride gave the ketone **199**, which was reduced with Evans-Tishchenko protocol to afford the β-hydroxy ketone **200**. Olefin cross-metathesis reaction of **200** with methyl acrylate proceeded selectively at the terminal olefin, giving rise to the α,β-unsaturated ester **201**, after BOM etherification. The crucial intramolecular oxa-Michael reaction was undertaken by methanolysis of the propionate group followed by DBU treatment in toluene at 100 °C to provide the 2,6-*cis*-substituted tetrahydropyran **202** in 53% yield (two steps). Hydrolysis of the ester moiety then afforded the carboxylic acid **203**. The coupling partner of **203**, the alcohol **204**, was prepared from (*R*)-epichlorohydrin in seven steps. Yamaguchi esterification of **203** with **204** provided the ester **205**, which underwent ring-closing metathesis under the influence of **G**-**II** and 1,4-benzoquinone [154] in toluene at 100 °C to furnish the macrocycle **206** in 85% yield. Hydrogenation/hydrogenolysis of **206** followed by Mitsunobu esterification of the derived alcohol **37** with the carboxylic acid **38** completed the total synthesis of neopeltolide (**4**). The present synthesis proceeded in a 13 linear-step sequence from *trans*-cinnamaldehyde (**196**).

### 6.4. Formal Synthesis by the Subhash Ghosh Group

**Figure 23:**
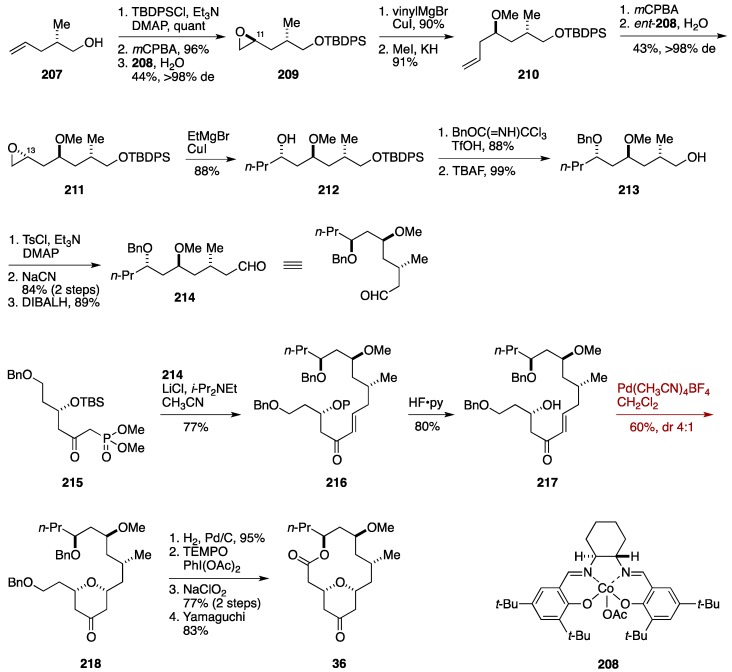
Subhash Ghosh formal synthesis of neopeltolide.

Subhash Ghosh and co-workers from Indian Institute of Chemical Technology reported a formal synthesis of neopeltolide, featuring a palladium-catalyzed intramolecular oxa-Michael addition for constructing the tetrahydropyran substructure [55]. Another feature of the Subhash Ghosh synthesis is the use of Jacobsen hydrolytic kinetic resolution methodology [155] for controlling the C11 and C13 stereogenic centers. The synthesis commenced with known alcohol **207**, prepared in two steps via an Evans asymmetric alkylation (Figure 23). After silylation, *m*CPBA epoxidation of the terminal olefin gave an epoxide as a mixture of diastereomers, whose hydrolytic kinetic resolution provided the epoxide **209** with >98% de. Addition of vinylmagnesium bromide in the presence of CuI and methylation of the resultant alcohol delivered the methyl ether **210**. *m*CPBA epoxidation of **210** was followed by hydrolytic kinetic resolution to afford the epoxide **211** with >98% de. Addition of EtMgBr/CuI gave the alcohol **212**, which was benzylated and then desilylated to produce the alcohol **213**. Tosylation, displacement with cyanide, and DIBALH reduction provided the aldehyde **214**. Horner-Wadsworth-Emmons reaction of **214** with the phosphonate **215** under Masamune-Roush conditions gave rise to the α,β-unsaturated ketone **216**. After removal of the silyl group, intramolecular oxa-Michael reaction was best performed by using Pd(CH_3_CN)_4_BF_4_ catalyst in CH_2_Cl_2_ at room temperature [156], affording the 2,6-*cis*-substituted tetrahydropyran-4-one **218** in 60% yield with 4:1 diastereoselectivity. After separation of the diastereomers, hydrogenolysis of the benzyl ethers, selective oxidation of the primary alcohol to the corresponding carboxylic acid, and Yamaguchi macrolactonization furnished the ketone **36**, thereby intersecting the Scheidt synthesis. The Subhash Ghosh formal synthesis was completed in 22 steps from a commercially available material.

Florence and Cadou reported their synthetic studies on neopeltolide, in which a transannular oxa-Michael reaction was planned as a key transformation [157]. Very recently, Boddy *et al.* reported the synthesis of a neopeltolide model compound by using a transannular oxa-Michael addition [158].

## 7. Synthesis of Tetrahydropyrans via Palladium-Catalyzed Intramolecular Alkoxycarbonylation

**Figure 24:**
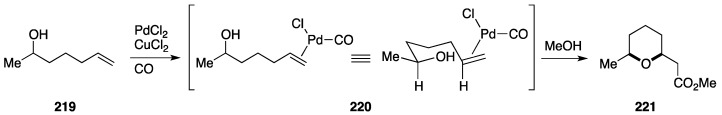
Palladium-catalyzed intramolecular alkoxycarbonylation.

Semmelhack *et al.* developed palladium-catalyzed intramolecular alkoxycarbonylation of alkenol derivatives, allowing access to a range of tetrahydropyran derivatives under mild conditions [159]. An example is given in Figure 24. The stereochemical consequence generally depends on the local structure of substrates and can be reasoned by the conformation of the intermediary palladium-complexed species [160].

### 7.1. Formal Synthesis by the She Group

**Figure 25:**
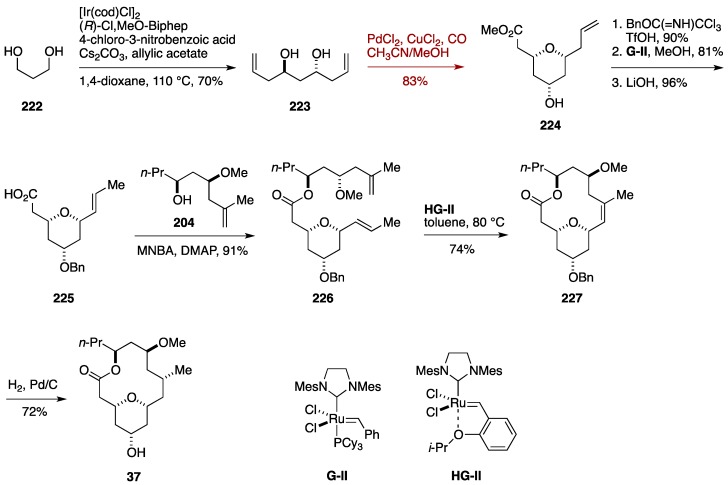
She formal synthesis of neopeltolide.

She *et al.* utilized a desymmetrizing palladium-catalyzed intramolecular alkoxycarbonylation for constructing the tetrahydropyran ring of neopeltolide (Figure 25) [52]. The synthesis began with Krische iridium-catalyzed double asymmetric carbonyl allylation [161,162] of 1,3-propanediol (**222**) to deliver the bis-homoallylic alcohol **223** in 70% yield. Desymmetrizing palladium-catalyzed intramolecular alkoxycarbonylation of **223** in the presence of PdCl_2_/CuCl_2_ in CH_3_CN/MeOH under CO atmosphere at room temperature afforded the 2,6-*cis*-substituted tetrahydropyran derivative **224** in 83% yield. Benzylation of **224** under acidic conditions was followed by the double bond transposition [163], and ensuing ester hydrolysis gave the carboxylic acid **225**. The macrocyclic skeleton was forged according to the second-generation Fuwa synthesis. Thus, Shiina esterification of **225** with the alcohol **204** provided the ester **226**, whose ring-closing metathesis using the second-generation Hoveyda-Grubbs catalyst (**HG**-**II**) [164] afforded the macrocycle **227**. Finally, hydrogenation/hydrogenolysis funished neopeltolide macrolactone **37**. The She formal synthesis was consisted of 12 linear steps from l-valinol via the alcohol **204**.

### 7.2. Total Synthesis of 9-Demethylneopeltolide by the Dai Group

**Figure 26:**
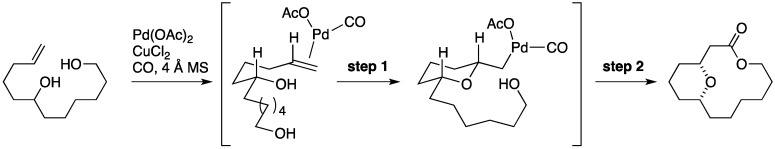
Palladium-catalyzed macrocyclic alkoxycarbonylation.

9-Demethylneopeltolide is a synthetic analogue of neopeltolide, originally reported by the Fuwa group [47]. This analogue showed potent antiproliferative activity against the P388 murine leukemia cell line. Dai and co-workers reported a total synthesis of 9-demethylneopeltolide, which involved an innovative use of palladium-catalyzed intramolecular alkoxycarbonylation for simultaneous construction of the tetrahydropyran ring and the macrocyclic skeleton [165]. Thus, Dai *et al.* envisioned that treatment of appropriately designed alkenediols, such as that shown in Figure 26, with Pd(OAc)_2_/CuCl_2_/CO undergoes an oxypalladation (step 1) to deliver the intermediary organopalladium species, which further participates in a macrocyclic alkoxycarbonylation (step 2) to afford tetrahydropyran-embedded macrocycles.

**Figure 27:**
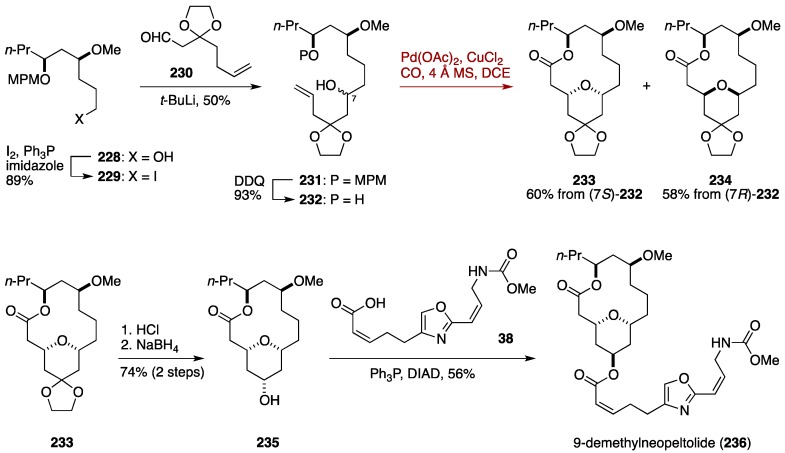
Dai total synthesis of 9-demethylneopeltolide.

The Dai synthesis of 9-demethylneopeltolide (**236**) started with iodination of known alcohol **228**, prepared in eight steps from ethyl 3-oxohexanoate, to give the iodide **229** (Figure 27). The iodide **229** was lithiated with *t*-BuLi and coupled with the aldehyde **230** to afford the adduct **231**, from which the MPM group was removed to provide the alcohol **232** as a 1:1 mixture of diastereomers. Macrocyclic palladium-catalyzed alkoxycarbonylation of **232** was achieved in the presence of Pd(OAc)_2_/CuCl_2_ and 4 Å molecular sieves in 1,2-dichloroethane at room temperature under CO atmosphere, giving a separable mixture of the macrocycles **233** and **234**. Hydrolysis of the acetal of **233** and subsequent NaBH_4_ reduction provided the alcohol **235**, which was coupled with the carboxylic acid **38** to afford 9-demethylneopeltolide (**236**).

## 8. Synthesis of Tetrahydropyrans via Transannular Oxymercuration

Oxymercuration is a classical method for hydration of alkenes and alkynes to give alcohols and ketones, respectively. This method can be applied to the synthesis of ethers by using an alcohol instead of H_2_O. Thus, oxymercuration of δ-alkenyl alcohols provides tetrahydropyran derivatives, after demercuration; addition of Hg(OAc)_2_ or its equivalent to an alkene forms an mercurinium ion intermediate, to which an *anti*-specific addition of an alcohol takes place to give an ether. Therefore, the stereochemical outcome depends on the diastereoselectivity of the mercurinium ion forming step.

### Formal Synthesis by the Sharma Group

**Figure 28:**
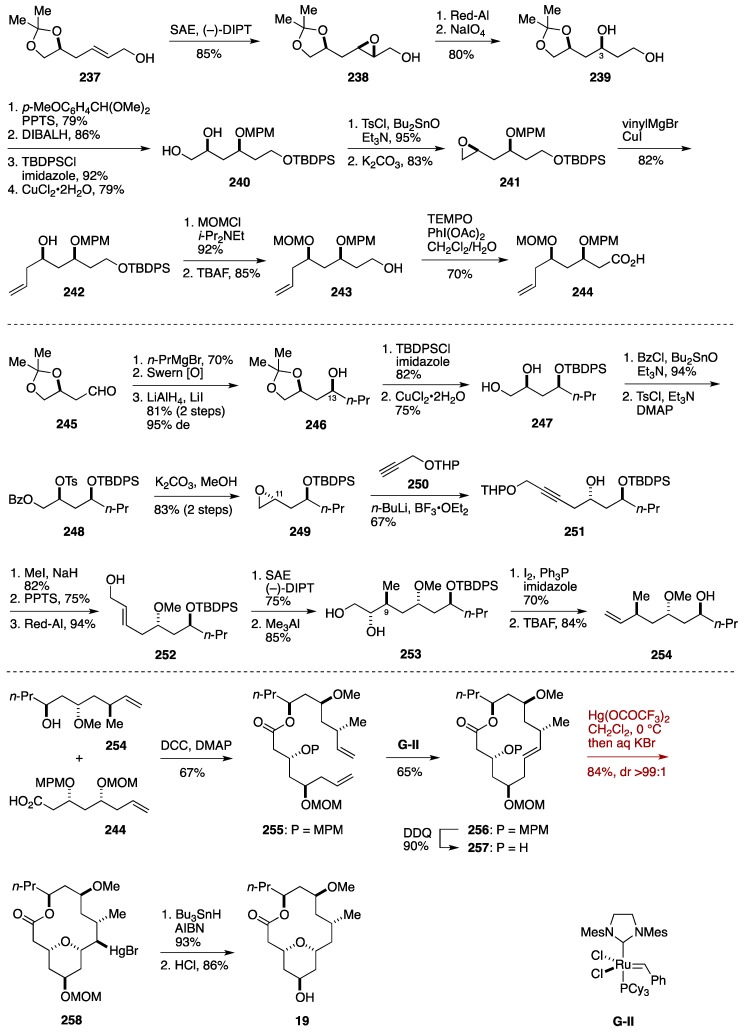
Sharma formal synthesis of neopeltolide.

The Sharma group synthesized two key fragments from L-malic acid and then forged the macrocyclic skeleton of neopeltolide by means of an esterification, a ring-closing metathesis, and a transannular oxymercuration (Figure 28) [53].

The synthesis of one of the key fragments, the carboxylic acid **244**, started with Sharpless asymmetric epoxidation of the allylic alcohol **237** [166] to give the epoxy alcohol **238**, which was regioselectively reduced with Red-Al [167] to deliver the 1,3-diol **239**, in which the C3 stereogenic center was appropriately installed. After a four step-sequence of protecting group manipulations [168], the resultant 1,2-diol **240** was transformed to the epoxide **241** via a tosylate [169]. The epoxide **241** was opened with vinylmagnesium bromide/CuI to give the homoallylic alcohol **242**, which was protected as its MOM ether and then desilylated to provide the alcohol **243**. This was oxidized to the carboxylic acid **244**.

The synthesis of another key fragment, the alcohol **254**, commenced with Grignard addition of *n*-propylmagnesium bromide to the aldehyde **245** [170], and the resultant alcohol was oxidized and then reduced with LiAlH_4_/LiI [171] to deliver the alcohol **246** to correct the configuration at the C13 stereogenic center. Silylation and acetonide cleavage gave the 1,2-diol **247**. Selective benzoylation of the primary alcohol and subsequent tosylation of the remaining alcohol led to the tosylate **248**. Exposure of **248** to basic conditions resulted in removal of the benzoyl group and spontaneous epoxide formation to deliver the epoxide **249**, setting the C11 stereogenic center. Alkynylation of **249** with the propargyl ether **250** under Yamaguchi conditions [172] gave the alcohol **251**. *O*-Methylation, cleavage of the THP ether, and stereospecific reduction of the alkyne provided the allylic alcohol **252**. Sharpless asymmetric epoxidation of **252** was followed by regioselective methylation of the resultant epoxy alcohol [173] to afford the 1,2-diol **253** with the C9 stereogenic center established. Treatment of **253** with I_2_, Ph_3_P, and imidazole [174] and subsequent desilylation delivered the alcohol **254**.

Esterification of **244** with **254** and subsequent ring-closing metathesis of the ester **255** led to the macrocycle **256**. After removal of the MPM group, the resultant alcohol **257** was exposed to Hg(OCOCF_3_)_2_ and then treated with aqueous KBr solution to afford the transannular product **258** in 84% yield with >99:1 diastereoselectivity. In contrast to the oxymercuration, iodoetherification of **257** using iodine or NIS resulted in a reversal of the diastereoselectivity. Compound **258** was reduced under radical conditions to furnish, after cleavage of the MOM ether, neopeltolide macrolactone **19**. The Sharma formal synthesis was completed in 24 linear steps from L-malic acid.

## 9. Synthesis of Tetrahydropyrans via Desymmetrizing Ring-Opening/Cross-Metathesis Cascade

**Figure 29:**
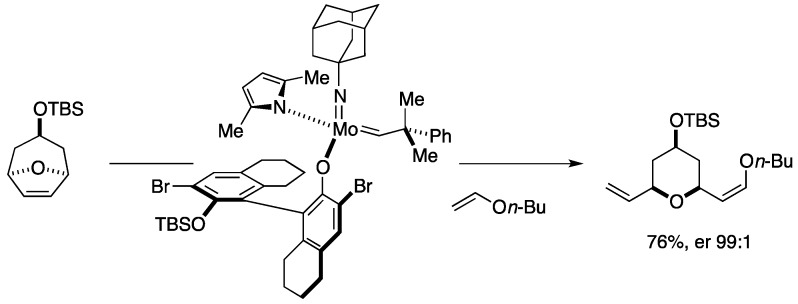
Desymmetrizing ring-opening/cross-metathesis.

Recent advances in the development of chiral stereogenic-at-molybdenum/ruthenium catalysts have made it possible to perform enantioselective olefin metathesis reactions of achiral and meso compounds to produce chiral compounds with high optical purity [175,176]. As shown in Figure 29, such a chiral stereogenic-at-molybdenum catalyst has been found to be effective for desymmetrizing ring-opening/cross-metathesis cascade reaction of meso-oxabicycles in the presence of *n*-butyl vinyl ether, giving rise to enantiomerically enriched, 2,6-*cis*-substituted tetrahydropyran derivatives [177].

### Total Synthesis by the Hoveyda Group

**Figure 30:**
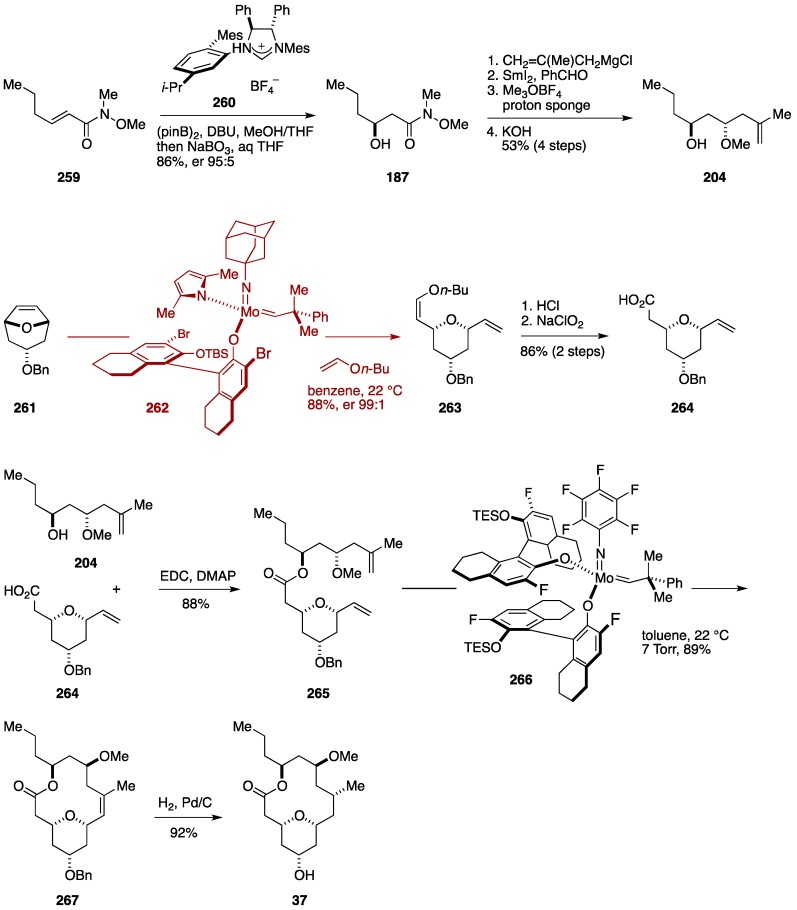
Hoveyda total synthesis of neopeltolide: Synthesis of neopeltolide macrolactone.

The most recent total synthesis of neopeltolide came from the Hoveyda group, in which their originally developed molybdenum and ruthenium catalysts were exploited in stereoselective olefin metathesis reactions (Figure 30). The Hoveyda synthesis started with enantioselective conjugate borylation [178] of the Weinreb amide **259**, prepared in one step from a commercially available material, followed by oxidative workup to give the β-alkoxy amide **187**. Addition of 2-methallylmagnesium chloride, Evans-Tishchenko reduction, *O*-methylation, and methanolysis delivered the alcohol **204**. Meanwhile, the synthesis of the coupling partner, the carboxylic acid **264**, began with desymmetrizing ring-opening/cross-metathesis cascade of meso-oxabicycle **261**, prepared in four steps from a commercially available material, with *n*-butyl vinyl ether by the action of the chiral molybdenum monoaryloxide pyrrolide catalyst **262** [175] to afford the 2,6-*cis*-substituted tetrahydropyran **263** [177] in 88% yield with 99:1 er. Hydrolysis of the enol ether moiety was followed by Pinnick oxidation of the resultant aldehyde gave the carboxylic acid **264**. Esterification of **264** with **204** led to the ester **265**. Ring-closing metathesis of **265** was performed under the influence of the chiral molybdenum bis(aryloxide) complex **266** [179] in toluene at 22 °C (7.0 Torr) to furnish the macrocycle **267** in 89% yield. Hydrogenation/hydrogenolysis of **267** then gave neopeltolide macrolactone **37**.

**Figure 31:**
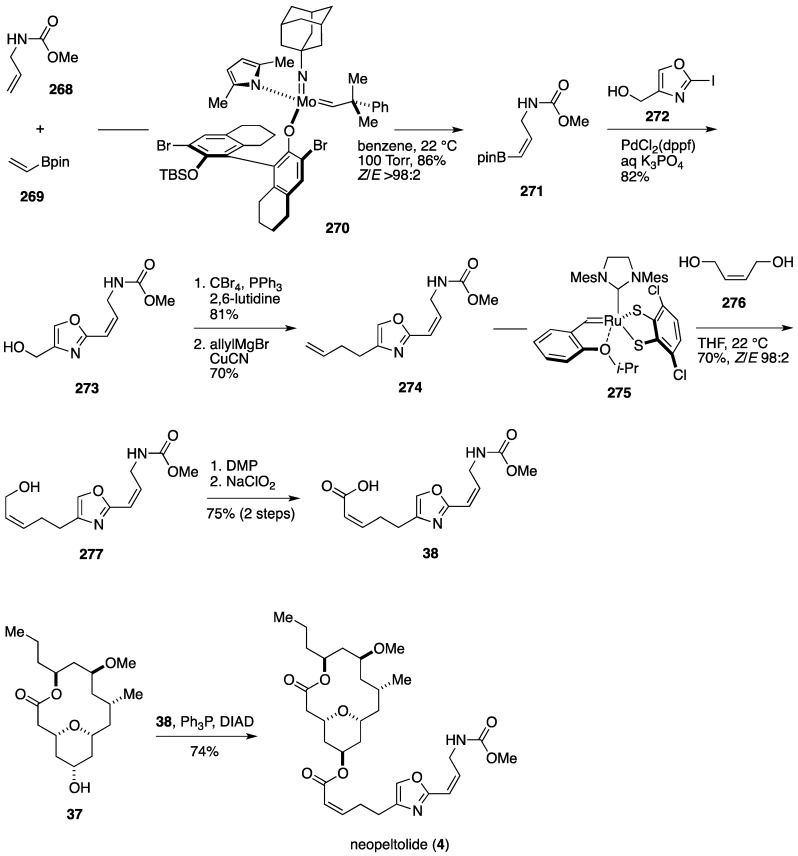
Hoveyda total synthesis of neopeltolide: Synthesis of oxazole-containing side chain and completion.

Hoveyda *et al.* also developed de novo synthesis of the oxazole-containing side chain of neopeltolide (Figure 31). The synthesis started from the allylic carbamate **268**. *Z*-Selective cross-metathesis of **268** with vinyl boronic acid pinacol ester (**269**) was achieved by using the chiral molybdenum monoaryloxide pyrrolide complex **270** [175] (benzene, 22 °C, 100 Torr) to give the vinyl boronate **271** in 86% yield (*Z*/*E* > 98:2). Suzuki-Miyaura reaction of **271** with the iodooxazole **272** delivered the alcohol **273**, which was brominated and then allylated to provide the olefin **274**. Olefin cross-metathesis of **274** with (*Z*)-2-buten-1,4-diol (**276**) in the presence of the ruthenium catechothiolate complex **275** [180] (THF, 22 °C) afforded the (*Z*)-allylic alcohol **277** in 70% yield. A two-stage oxidation of **277** then delivered the carboxylic acid **38**. Finally, Mitsunobu esterification of **37** with **38** completed the total synthesis of neopeltolide (**4**). The Hoveyda synthesis represents the most concise synthesis of neopeltolide, showcasing the power of chiral stereogenic-at-molybdenum catalysts for asymmetric synthesis of complex molecules.

## 10. Conclusions

More than twenty reports have described the total/formal syntheses of neopeltolide and its analogues so far, and a variety of synthetic strategies for constructing the tetrahydropyran ring of this natural product have been demonstrated therein. These synthetic efforts have shown that versatile bond-forming reactions that do not require multistep pre-functionalization of substrates are key to minimize the step-count and to maximize the synthetic efficiency. It has also been shown that multiple bond-forming cascade and desymmetrization are key strategic concepts for facilitating rapid construction of tetrahydropyran derivatives. It is more than likely that these lessons have implications for practical total synthesis of complex macrolide natural products in general.

Because of its highly potent antiproliferative activity against several human cancer cell lines, neopeltolide may represent an intriguing anticancer lead compound. However, the molecular basis of the antiproliferative activity of this natural product has not been fully understood, and this point needs to be addressed before any development. Given the “multi-ligandability” of many natural products [10], chemical biology investigations using designed molecular probes and/or affinity matrices, now available through total synthesis, would shed light on the mechanism(s) by which neopeltolide exerts its potent activity.

## Abbreviations

The following abbreviations are used in this manuscript:

9-BBN	9-borabicyclo[3.3.1]nonyl
acac	acetylacetone
AIBN	2,2′-azobis(isobutyronitrile)
BINAP	2,2′-bis(diphenylphosphino)-1,1′-binaphthyl
BINOL	1,1′-bi-2-naphthol
Biphep	2,2′-bis(diphenylphosphino)-1,1′-biphenyl
Bn	benzyl
BOM	benzyloxymethyl
Bz	benzoyl
cod	1,5-cyclooctadienyl
Cp*	1,2,3,4,5-pentamethylcyclopentadienyl
CSA	10-camphorsulfonic acid
Cy	cyclohexyl
DBB	4,4′-di-*t*-butylbiphenyl
DBU	1,8-diazabicyclo[5.4.0]undec-7-ene
DCC	dicyclohexylcarbodiimide
DCE	1,2-dichloroethane
DDQ	2,3-dichloro-5,6-dicyanobenzoquinone
DEAD	diethyl azodicarboxylate
DET	diethyl tartrate
DIAD	diisopropyl azodicarboxylate
DIBALH	diisobutylaluminum hydride
DIPT	diisopropyl tartrate
DMAP	4-dimethylaminopyridine
DMF	*N*,*N*-dimethylformamide
DMP	Dess-Martin periodinane
DMSO	dimethylsulfoxide
dppf	1,1′-bis(diphenylphosphino)ferrocene
DTBMP	2,6-di-*t*-butyl-4-methylpyridine
DVDS	1,3-divinyl-1,1,3,3-tetramethyldisiloxane
EDC	1-(3-dimethylaminopropyl)-3-ethylcarbodiimide hydrochloride
HMPA	hexamethylphosphoramide
HOBt	1-hydroxybenzotriazole
IBX	2-iodoxybenzoic acid
Ipc	isopinocampheyl
(*S*,*R*)-Josiphos	(*S*)-1-[(*R*_p_)-2-(diphenylphosphino)ferrocenyl]ethyldicyclohexylphosphine
KHMDS	potassium hexamethyldisilazide
LDA	lithium diisopropylamide
*m*CPBA	*m*-chloroperbenzoic acid
Mes	mesityl
MNBA	2-methyl-6-nitrobenzoic anhydride
MOM	methoxymethyl
MPM	*p*-methoxyphenylmethyl
MS	molecular sieves
NMO	*N*-methylmorpholine *N*-oxide
pin	pinacol
PPTS	pyridinium *p*-toluenesulfonate
Ra/Ni	Raney-Nickel catalyst
Red-Al	sodium bis(2-methoxyethoxy)aluminum hydride
SAE	Sharpless asymmetric epoxidation
Synphos	[(5,6),(5′,6′)-bis(ethylenedioxy)biphenyl-2,2′-diyl]bis(diphenylphosphine)
TBAF	tetra-*n*-butylammonium fluoride
TBDPS	*t*-butyldiphenylsilyl
TBS	*t*-butyldimethylsilyl
TEMPO	2,2,6,6-tetramethylpiperidin-1-oxyl radical
TES	triethylsilyl
Tf	trifluoromethanesulfonyl
TFA	trifluoroacetic acid
TMS	trimethylsilyl
Ts	*p*-toluenesulfonyl
